# Regulation of ENaC-Mediated Sodium Reabsorption by Peroxisome Proliferator-Activated Receptors

**DOI:** 10.1155/2010/703735

**Published:** 2010-06-14

**Authors:** Tengis S. Pavlov, John D. Imig, Alexander Staruschenko

**Affiliations:** ^1^Department of Physiology, Medical College of Wisconsin, Milwaukee, WI 53226, USA; ^2^Department of Pharmacology and Toxicology, Medical College of Wisconsin, Milwaukee, WI 53226, USA; ^3^Cardiovascular Research Center, Medical College of Wisconsin, Milwaukee, WI 53226, USA; ^4^Kidney Disease Center, Medical College of Wisconsin, Milwaukee, WI 53226, USA

## Abstract

Peroxisome proliferator-activated receptors (PPARs) are members of a steroid hormone receptor superfamily that responds to changes in lipid and glucose homeostasis. Peroxisomal proliferator-activated receptor subtype *γ* (PPAR*γ*) has received much attention as the target for antidiabetic drugs, as well as its role in responding to endogenous compounds such as prostaglandin J_2_. However, thiazolidinediones (TZDs), the synthetic agonists of the PPAR*γ* are tightly associated with fluid retention and edema, as potentially serious side effects. The epithelial sodium channel (ENaC) represents the rate limiting step for sodium absorption in the renal collecting duct. Consequently, ENaC is a central effector impacting systemic blood volume and pressure. The role of PPAR*γ* agonists on ENaC activity remains controversial. While PPAR*γ* agonists were shown to stimulate ENaC-mediated renal salt absorption, probably via Serum- and Glucocorticoid-Regulated Kinase 1 (SGK1), other studies reported that PPAR*γ* agonist-induced fluid retention is independent of ENaC activity. The current paper provides new insights into the control and function of ENaC and ENaC-mediated sodium transport as well as several other epithelial channels/transporters by PPARs and particularly PPAR*γ*. The potential contribution of arachidonic acid (AA) metabolites in PPAR-dependent mechanisms is also discussed.

## 1. Introduction

Peroxisome proliferator-activated receptors (PPARs) are ligand-activated transcription factors belonging to a nuclear hormone receptor superfamily containing three isoforms (alpha, beta/delta, and gamma). The name peroxisome proliferator-activated receptor derives from the ability of the first member of the family identified, PPAR*α*, to respond to various compounds that induce peroxisome proliferation. This function is not shared by other members of the PPAR family. Instead, they have emerged as major regulators of various aspects of lipid metabolism and metabolic control [1–3]. PPAR-*β*/*δ* (NR1C2) is ubiquitously expressed in many tissues, especially in the brain; however, its function has yet to be clearly defined. 

This paper will focus on two isoforms: PPAR*γ* and to less extent PPAR*α*. Their role has been increasingly recognized to be important in hypertension, metabolic disorders and cardiovascular diseases. Furthermore nuclear receptors including PPAR*γ* are promising targets for drug development. Functioning as transcription factors they control cellular processes at the level of gene expression. Their modulation of nuclear receptor activity produces selective alterations in downstream gene expression. PPARs regulate gene transcription by two mechanisms: (1) transactivation, a DNA-dependent mechanism, which involves binding to PPAR response elements (PPRE) of target genes and heterodimerization with the retinoid X receptor (RXR); (2) transrepression, a process that may explain the contradictory of actions of PPARs. Transrepression involves interfering with other transcription-factor pathways in a DNA-independent way [1]. 

Various fatty acids serve as endogenous ligands for PPARs: prostaglandin J_2_ is an endogenous PPAR*γ* ligand, whereas PPAR*α* is activated by cytokines. After ligand binding, PPARs undergo specific conformational changes that allow for the recruitment of one coactivator protein or more. PPARs function as an obligate heterodimer with RXRs and recognize PPRE located in the promoter region of target genes [4]. In addition, PPAR activators were shown to inhibit the activation of inflammatory response genes by negatively interfering with the nuclear factor-*κ*B (NF-*κ*B), STAT and AP-1 signaling pathways [5]. These characteristics, combined with PPARs involvement in significant diseases, make nuclear receptors a key target for the development of disease-specific therapies.

## 2. PPARs and Their Role in Hypertension and Fluid Retention

Insulin-sensitizing drugs thiazolidinediones (TZDs) are known as PPAR*γ*-agonists, and hypolipidemic drugs fibrates are known as PPAR*α* ligands. Agonists of PPAR*γ* and PPAR*α* are currently approved for use in treating type 2 diabetes and dyslipidemia, respectively.

PPAR*α* is expressed predominantly in the liver, heart, muscle, and vascular wall. Sato and colleagues generated isoform-specific anti-PPAR antibodies to determine the localization of these proteins in the rat kidney [6]. Regarding PPAR*α*, it was widely expressed along the nephron segments (proximal and distal tubules, the loop of Henle, and medullary collecting ducts) glomeruli, and intima/media of renal vasculatures. Previous studies reported that PPAR*α* expression was mainly localized to the proximal tubules [7–9]. Based on the prominent distribution of PPAR*α* in the kidney, PPAR*α* is likely to affect renal salt transport. However, it is important to note that tissue expression of all three PPAR isoforms is likely to vary under differing physiological and/or pathological conditions. 

Interestingly, studies using PPAR*α* ^−/−^ mice have produced conflicting findings in regard to its role in blood pressure regulation [10]. Deletion of PPAR*α* has been shown to significantly increase [11], decrease [12] and have no significant effect [13] on systolic blood pressure compared with wild-type controls. Further studies suggest that PPAR*α*  participates in pressure natriuresis and affects Na^+^ transport via amiloride- and thiazide-sensitive mechanisms. Despite defective fatty acid oxidation, PPAR*α* null mice are not hypertensive but develop salt-sensitive hypertension [14]. The observation that clofibrate improved pressure natriuresis [15] and PPAR*α* activation inhibited ion transport in the isolated proximal tubules [16], gives credence to the notion that PPAR*α* may be involved in the salutary effects of the kidney. However, a role of PPAR*α* in ENaC-mediated sodium reabsorption is still unclear. 

Troglitazone was the first TZD which was approved in January 1997 as a glucose-lowering therapy because it enhances insulin sensitivity in patients with type 2 diabetes [17]. Due to hepatotoxicity, it has since been replaced by PPAR*γ*-agonists rosiglitazone and pioglitazone [1]. However, both rosiglitazone and pioglitazone have been associated with increased development of edema in clinical trials. Non-insulin-dependent diabetes mellitus with associated insulin insensitivity and reactive hyperinsulinemia is often complicated by hypertension. Increased blood pressure could in part be due to volume expansion resulting from improper avid Na^+^ reabsorption by the kidney. Insulin increases Na^+^ reabsorption in the distal renal nephron; likely by targeting ENaC localized to the luminal membrane of principal cells. The highest incidence of edema has been reported when TZDs are utilized in combination with insulin. 

In addition to peripheral edema, reports have described pulmonary edema and heart failure associated with thiazolidinedione therapy. In all such reports, patients failed to respond to diuretics during use of TZDs. Clinical improvement ensued only after discontinuation of TZD therapy [18, 19]. A water-electrolyte imbalance observed in these patients led researchers to investigate PPARs participation in kidney function. Laboratory studies have demonstrated that rosiglitazone treatment does not affect blood pressure or angiotensin-II (Ang II) production by mesangial cells and plays a renoprotective role in Sprague-Dawley rats [20]. Moreover, TZDs normalized expression of angiotensin converting enzyme-1 in obese Zucker rats as well as lowered blood pressure and angiotensin-II synthesis in spontaneously hypertensive rats on a high-salt diet with glycerol-induced acute renal failure [21, 22].

It has been proposed that TZDs may be useful in the prevention and/or treatment of hypertension, particularly when it is associated with insulin resistance or diabetes mellitus. TZDs attenuate the development of hypertension, normalize cell growth, and improve endothelial dysfunction induced by (Ang II). In addition TZDs prevented upregulation of Ang II type 1 receptors, cell cycle proteins, and proinflammatory mediators [23]. It has been suggested that the apparent hypotensive effects of PPAR*γ* agonists in this model may result from down regulation of the Ang II type 1 receptor [10, 24]. Moreover, PPAR*γ* was identified as an intracellular mediator involved in the upregulation of renin transcription [25]. Using PPAR*γ*-knockdown in juxtaglomerular cells (RC-PPAR*γ*fl/fl mice), increased renin production was found even though arterial pressure was indistinguishable in these mice compared to wild-type animals [26]. 

Mouse models that disrupt PPAR*γ* specifically in the collecting ducts (CDs) in the kidney have been used to evaluate a role of PPAR*γ* in fluid retention. Guan et al. demonstrated that deletion of CD PPAR*γ* decreased renal Na^+^ avidity and increased plasma aldosterone. Mice treated with TZDs experience early weight gain from increased total body water. Moreover, weight gain was blocked by the diuretic amiloride [27]. Similarly, Zhang et al. found that mice with CD-specific knockout of PPAR*γ* were resistant to the rosiglitazone-induced increase in body weight and plasma volume expansion found in control mice expressing PPAR*γ* in the CD [28]. The role of PPAR*γ* in regulation of sodium transport in the kidney will be further discussed below.

## 3. PPARs and Arachidonic Acid Metabolites

Natural and synthetic ligands bind to PPARs, resulting in a conformational change and activation of PPAR. The identity of the biological ligands for PPARs remains unresolved and is an area of active investigation. The PGD_2_ metabolite, 15-deoxy-Δ^12,14^-prostaglandin J_2_ (15d-PGJ_2_), was the first endogenous ligand for PPAR*γ* to be discovered. Although 15d-PGJ_2_ is the most potent natural ligand of PPAR*γ*, the extent to which its effects are mediated through PPAR*γ* *in vivo* remains to be determined. It is unlikely that 15d-PGJ_2_ is present at sufficient levels *in vivo* to be a biologically significant PPAR ligand. Furthermore, it is possible that ligands for PPARs have different potency and efficacy and may be distinct in different tissues. 

The function of PPARs is modified by the precise shape of their ligand-binding domain; which is induced by ligand binding as well as a number of coactivators and corepressor proteins. It is well known that endogenous ligands for the PPAR*γ* include free fatty acids and  eicosanoids. Furthermore, the localization of CYP4A and PPAR*γ* proteins as well as the inducibility of Cyp4A expression and activity by PPAR*γ* agonists have been determined in the rat kidney [29]. Moreover, the effect of PPAR*γ* agonists on the Cyp4A pathway was recently demonstrated and provided experimental evidence that pioglitazone down regulates Cyp4A leading to sodium retention [30]. Treatment of hypertensive *Cyp4a10*(-/-) mice with Wyeth 14,643, (a selective PPAR*α* ligand), up-regulated renal Cyp2c44 expression, increased urine epoxyeicosatrienoic acid (EET) concentrations, and normalized the animals systemic blood pressure [31, 32]. Thus, it is possible that eicosanoids and free fatty acids might be involved in PPARs-regulated changes in fluid retention.

Multiple factors are presumed to regulate renal sodium handling including the renin-angiotensin system (RAS), nitric oxide (NO), and prostaglandins. Recent studies have demonstrated that PPAR*γ* ligands enhance NO production through the increased expressions of NO synthase (NOS) in the kidneys of obese rats, thus shifting the sodium balance to increased excretion [33]. In addition, PPAR*γ* is reported to induce cyclooxygenase-2 (COX-2), an enzyme producing PGE_2_ and PGI_2_ in several cell lines [34], which also could favor sodium excretion. 

Arachidonic acid (AA) is primarily metabolized by cytochrome *P*-450 (CYP) enzymes to 19- and 20-hydroxyeicosatetraenoic acids (19- and 20-HETE) and EETs (5,6-, 8,9-, 11,12-, and 14,15-EET) and these compounds play critical roles in the regulation of renal, pulmonary and cardiac function [35]. The expression of CYP 4A (CYP4A) isoform was determined in cortical collecting ducts (CCDs) [36]. Similarly, *Cyp2C* isoforms are expressed in the CCD [37–39]. It was previously shown that AA significantly decreases ENaC activity in CCD cells. Adenosine inhibits ENaC activity by stimulation of the A_1_ adenosine receptor in the CCD and the effect of adenosine is mediated by 11,12-EET [38, 40, 41]. 11,12-EET has also been shown to mediate AA-induced inhibition of 18 pS basolateral K^+^ channels [42] and activation of BK channels in the apical membrane of the CCD [39]. 20-HETE and EETs have actions on the thick ascending limb (TAL) cells that would decrease sodium reabsorption. 20-HETE production is involved in reducing the activity of the apical 70 pS K^+^ channels in the medullary TAL (mTAL) [43, 44]. AA inhibits 50 pS K^+^ channels in the basolateral membrane of the mTAL mainly through CYP-dependent metabolities of AA [45]. Recently it was demonstrated that AA inhibits the 10 pS chloride channel in the basolateral membrane of the mTAL and that the effect is mediated by 20-HETE [46]. Thus, AA and its metabolites are involved in the regulation of various ion channels in the kidney. Moreover, a dysfunctional *Cyp4a10* gene causes a type of hypertension that is dietary salt sensitive and associated with alterations in the gating activity of ENaC [31]. The above observations allow the speculation that TZDs might suppress specific AA metabolite production, therefore reducing sodium excretion.

## 4. PPARs and ENaC

The regulation of sodium reabsorption is one of the most important questions in the area of kidney physiology. ENaC represents the rate limiting step for Na^+^-reabsorption across many epithelia, including those in the distal nephron, lungs and colon, and as such plays a central role in salt homeostasis and blood pressure control [47, 48]. ENaC-mediated sodium transport plays a pivotal role in homeostasis of epithelial kidney tissues and other organs. Abnormalities in ENaC function have been linked to disorders of total body Na^+^ homeostasis, blood volume, blood pressure, and lung fluid balance [49, 50]. Despite several reports testing regulation of ENaC by PPAR*γ*, the role of PPAR*γ* to regulate ENaC has not yet been fully resolved and remains controversial. Here, we summarize the current knowledge regarding involvement of PPARs in regulation of this channel.

Guan et al. found that treatment with rosiglitazone and pioglitazone causes body fluid accumulation in C57BL/6J mice within a span of days. Pretreatment of mice with amiloride, a selective inhibitor of ENaC, completely blocked weight gain produced by subsequent treatment with pioglitazone. To establish whether CD PPAR*γ* expression is required for TZD-associated weight gain, mice were generated that selectively lack PPAR*γ* in their CDs. This deletion prevented pioglitazone-induced fluid retention, decreased renal Na^+^ avidity and increased plasma aldosterone. Treating cultured CDs with TZDs increased amiloride-sensitive Na^+^ absorption and *Scnn1g* mRNA (encoding ENaC *γ*-subunit) expression through a PPAR*γ*-dependent pathway [27]. Independently, Zhang and colleagues showed that CD-specific deletion of PPAR*γ* blocks TZD-induced fluid retention in mice and that rosiglitazone stimulated sodium transport in primary cultures of CDs cells expressing PPAR*γ* but not in cells lacking this receptor [28]. Plasma volume expansion, decreased urine volume and sodium excretion were also observed in Sprague-Dawley rats treated with TZDs rosiglitazone and farglitazar [51, 52].

It was proposed that PPAR*γ* activators may increase renal Na^+^ reabsorption by stimulating ENaC and Serum- and Glucocorticoid-Regulated Kinase 1 (SGK1). The CCD is the major segment mediating the sodium-retaining effects of aldosterone, primarily through the upregulation of ENaC activity. The early effect of aldosterone on ENaC-*α* translocation is believed to be regulated by SGK1; as SGK1 expression is rapidly induced by aldosterone and strongly stimulates ENaC activity [54, 55]. PPAR*γ* is abundant in native CCD [9]. PPAR*γ* is also highly expressed in human renal CCD [56], the A6, M1 and mpkCCD_c14_ cells [57]. Splice variants of the human PPAR*γ* (PPAR-*γ*_1_ and PPAR-*γ*_2_) have been identified [58]. It was shown that PPAR-*γ*_1_, but not PPAR-*γ*_2_, was expressed along the nephron segments predominantly in the cortex [6].

Hong et al. have previously shown that SGK1 activity in a human CCD cell line is stimulated by treatment with PPAR*γ* agonists. These changes are paralleled by an increase in SGK1 mRNA which may be abolished by pretreatment with a specific PPAR*γ* antagonist. An increase in SGK1 mRNA may also lead to increased levels of cell surface ENaC*α* [56]. PPAR*γ* agonists have been proposed to increase renal Na^+^ reabsorption by stimulating SGK1 and ENaC [59]. Artunc et al. also have demonstrated that SGK1 contributes to the volume retention during treatment with pioglitazone using mice lacking SGK1 (sgk1–/–); however, SGK1 did not fully account for this effect [54]. 

Increasing evidence suggest that PPAR*γ* agonists do not directly alter ENaC activity or ENaC-mediated sodium reabsorption. Nofziger et al. first demonstrated that activation of PPAR*γ* with either pioglitazone or the more potent GW7845 does not directly enhance basal or insulin-stimulated Na^+^ transport via ENaC in the A6, M-1 or mpkCCD_c14_ cell lines. No change in SGK1 expression levels were found in mpkCCD_c14_ cells after 18–24 hrs treatment with PPAR*γ* agonists [57]. Similar to this study, we demonstrated no changes or even a slight decrease in basal sodium or insulin-stimulated reabsorption in mpkCCD_c14_ cells in response to different PPAR*γ* agonists including: pioglitazone, rosiglitazone, troglitazone and 15d-PGJ_2_. Moreover rosiglitazone has no effect on ENaC current density when the channel was reconstituted in CHO cells [53]. Wilson and colleagues have also recently confirmed our observations that rosiglitazone and pioglitazone had no discernible effect on transepithelial Na^+^ absorption in unstimulated or insulin-stimulated distal nephron cells and distal airway epithelial cells. Both TZDs also failed to alter cellular SGK1 activity [60]. 

Furthermore, Vallon and colleagues utilized mice with conditionally inactivated *α*-ENaC (Scnn1a^loxloxCre^) in CDs to test the role of ENaC in TZD-induced fluid retention. Interestingly, increased body weight, plasma volume, and fluid content of abdominal fat pads were observed both in control (Scnn1a^loxlox^) and Scnn1a^loxloxCre^ animals after rosiglitazone treatment. However, the treatment did not significantly alter the renal expression of ENaC subunits or Na-Cl cotransporter (NCC) in both mouse lines [61]. Similar, Khan et al. had earlier also demonstrated that renal cortical abundance of NCC was not significantly changed in obese Zucker rats after rosiglitazone treatment [62]. Vallon et al. proposed that TZDs-induced fluid retention and weight gain are mediated by nonselective cation channels (NSC) in inner medullary CD (IMCD) and ENaC-mediated Na^+^ reabsorption in CDs is not critical for this effect. According to single-channel data, a TZD diet did not affect ENaC activity in freshly isolated CCDs of wild-type mice [61]. These data strongly argue against a primary and critical role of ENaC in TZD-induced fluid retention. On the other hand, this study does not explain TZD-dependent increase of *γ*-ENaC mRNA expression [27] or cell surface expression measured with cell surface biotinylation of *α*-ENaC after 4 hrs and *α*-ENaC mRNA after 24 hrs treatment with TZDs in human CCD [56]. Furthermore, as proposed by Rubera et al., CD-specific inactivation of *α*-ENaC does not impair sodium balance [63]. When *α*-ENaC expression was deleted selectively from the CDs, leaving intact ENaC expression in the renal connecting tubule and nonrenal tissues, the mice were viable and exhibited only a very mild phenotype with little or no inability to maintain fluid homeostasis in the face of salt or water restriction [63]. 

**Figure 1:**
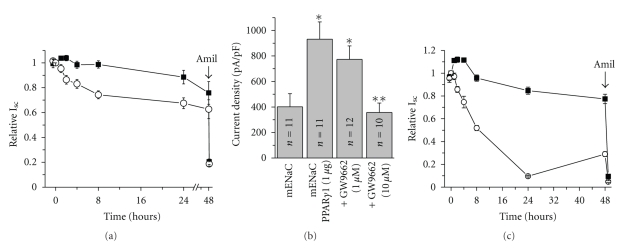
Effect of PPAR*γ* antagonists on amiloride-sensitive current. (a) Time course of decreases in relative Na^+^ transport in response to PPAR*γ* inhibition with GW9662 (10 *μ*M). mpkCCD_c14_ cells were serum-starved overnight. PPAR*γ* antagonist (circles) and vehicle (squares) were added at time 0 and current was normalized to starting level. Amiloride (10 *μ*M; arrow) was added to the apical membrane at the end of experiment. The numbers of experiments are 24 and 26 for GW9662 and vehicle, respectively. (b) Summary graph of ENaC current density in CHO cells expressing mENaC subunits alone or coexpressed with PPAR*γ*_1_ in the absence and presence of pretreatment with GW9662 (1 and 10 *μ*M). The number of experiments is shown. *P < .05, versus mENaC alone; **P < .05, versus mENaC + PPAR*γ*_1_. (c) Time course for T0070907-dependent decreases in relative Na^+^ transport across monolayers of mpkCCD_c14_ principal cells. The numbers of experiments are 9 and 6 for control and cells treated with T0070907 (50 *μ*M), respectively. All other conditions are the same as in (a). Some results represented here were previously published in a different format [53].

Our recent findings have shown that two PPAR*γ* antagonists, T0070907 and to a lesser extent GW9662, decrease Na^+^ reabsorption across mpkCCD_c14_ principal cells. Furthermore, pretreatment of monolayers with T0070907 diminished the insulin-stimulated sodium transport. Coexpression of PPAR*γ*_1_ enhances ENaC activity when all three channel subunits and PPAR*γ*_1_ are reconstituted in CHO cells (Figure 1) [53]. Thus, our data using PPAR*γ* antagonists are consistent with the idea that PPAR*γ* activity is important for maintaining basal and insulin-dependent transepithelial Na^+^ transport and ENaC activity. 

There are no studies showing acute effect of TZDs on ENaC-mediated sodium reabsorption. Thus, most likely TZDs affect epithelial transport through only genomic mechanisms. In transrepression, PPAR*γ* can repress gene transcription by negatively interfering with other signal-transduction pathways, such as NF-*κ*B signaling pathway, in a DNA-binding-independent manner [1]. The RXR is the required heterodimer partner of PPARs. It was previously shown that RXR*α* mRNA was present predominately in proximal convoluted tubule (PCT) and IMCD, whereas RXR*β* was present in all nephron segments examined except mTAL [9]. RXR, which interacts with the PPARs, is activated by 9-cis retinoic acid. When combined as a PPAR:RXR heterodimer, the PPAR ligands and 9-cis retinoic acid act synergistically on PPAR responses. It is still not clear whether TZDs regulate ENaC activity and ENaC-mediated sodium transport in the presence of 9-cis retinoic acid. 

**Figure 2:**
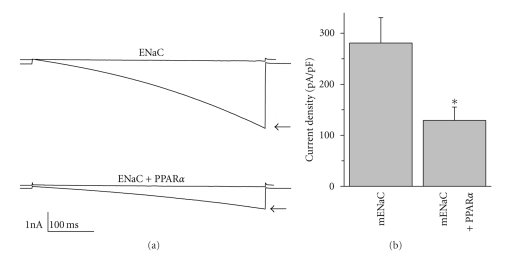
PPAR*α* inhibits ENaC when overexpressed in CHO cells. (a) Overlays of typical macroscopic current traces before (*arrow*) and after 10 *μ*M amiloride from voltage-clamped CHO cells transfected with mENaC alone (*top*) and with PPAR*α*. Currents were elicited by voltage ramping from 60 mV down to −100 mV (holding potential is 40 mV). (b) Summary graph of ENaC activity in CHO cells when all three mENaC subunits are expressed in the absence and presence of PPAR*α*. The numbers of experiments are 9 and 7 for control and cells overexpressed with PPAR*α* cDNA (1 *μ*g), respectively.

To date there are no reports for the involvement of PPAR*α* in regulation of ENaC activity. Our recent investigation surprisingly revealed a suppressive influence of PPAR*α* on amiloride-sensitive current density when coexpressed with ENaC subunits in CHO cells (Figure 2). Figure 2(a) shows overlays of ENaC currents from typical whole cell voltage clamp experiments elicited by standard voltage ramps before and after amiloride (10 *μ*M). Currents are from cells expressing all three mouse ENaC subunits (0.3 *μ*g each) alone or coexpressed with PPAR*α* (1 *μ*g). Whole-cell macroscopic current recordings of ENaC reconstituted in CHO cells were made under voltage-clamp conditions using standard methods [64–66]. Co-expression of PPAR*α* together with ENaC significantly decreased amiloride-sensitive current density from 281 ± 50 to 129 ± 26 pA/pF (Figure 2(b)). Although these data provide initial evidence it is not clear how PPAR*α* is involved in regulation of ENaC and ENaC-mediated sodium transport and requires additional investigations.

To place these observations in the context with those previously reported by others, our results appear to be most supportive of the idea that ENaC is involved in PPAR*γ*-regulated changes in fluid retention. However, possible mechanisms underlining the regulation of ENaC via PPAR*γ* remain obscure and require additional studies. Furthermore, the potential role of PPAR*α* and RXR in regulation of sodium transport needs to be clarified.

## 5. Other Targets of PPAR

Several additional studies argue against the notion that TZD-induced edema formation in CCDs is due to direct renal salt and water retention. Consequently, several mechanisms have been proposed. As discussed above, patch-clamp studies in primary mouse IMCD cells detected a NSC that was upregulated by pioglitazone [61]. Moreover, electrophysiological studies of cultured IMCD cells recently revealed that following exposure to rosiglitazone, amiloride-sensitive short-circuit current was unchanged at 24 hrs but was significantly decreased at 48 hrs. Along this line mRNA expression of all three ENaC subunits exhibited a trend of reduction at 24 hrs and a nearly 50% reduction at 48 hrs. Despite ENaC inhibition, transepithelial resistance was significantly reduced, suggesting an alternative route of increased ion transport. However, this effect was blunted in the IMCD cells derived from mice lacking PPAR*γ*. It has been proposed that rosiglitazone-treatment of primary IMCD cell monolayers exhibit increased paracellular Cl^−^ flux, and to a lesser extent, paracellular Na^+^ flux [67]. 

In addition various epithelial transport proteins may contribute to TZD-induced reabsorption in the nephron. The proximal tubule is known to mediate more than half of total tubular sodium and water reabsorption [68]. Saad and others have recently shown that PPAR*γ* agonists enhance the expression of NHE3 (Na^+^/H^+^ exchanger), AQP1 and AQP7 water channels in human proximal tubule cells through SGK1-dependent pathways [69]. Song and colleagues noticed that rosiglitazone increased whole kidney protein abundance of the *α*-1 subunit of Na^+^/K^+^-ATPase, Na-K-2Cl contransporter (NKCC2), NHE3, AQP 2 and 3, and NOS [51]. 

Several investigations have focused on the role of PPAR*γ* as a regulator of chloride transport through cAMP-regulated chloride channel (cystic fibrosis transmembrane regulator, CFTR). It was shown that PPAR*γ* expression is altered in tissue lacking CFTR. PPAR*γ* expression in cftr-/- mice is downregulated at the RNA and protein levels and its function diminished [70]. These changes may be related to the loss of function of CFTR and may be relevant to the pathogenesis of metabolic abnormalities associated with cystic fibrosis. Recently Nofziger et al. demonstrated that PPAR*γ* agonists inhibit vasopressin-mediated anion transport in the MDCK-C7 cells. The PPAR*γ* agonist-induced downregulation of anion secretion is the result of decreased CFTR expression and suggests a primary role for inhibition of CTRF in the development of water and electrolyte imbalance during PPAR*γ* agonist therapy [71–73]. Similar TZD-mediated effects on Cl^−^ flux has been demonstrated in mouse intestinal epithelia [74]. PPAR*γ* agonists have been considered for treatment of cystic fibrosis on the basis of their anti-inflammatory activities [75]. Importantly, Harmon and colleagues have recently established that additional parameters, such as metabolites of AA should be considered in the design of clinical trials [76]. Experimental studies have shown that colonic epithelial cells and whole lung tissue from *Cftr*-deficient mice show a defect in PPAR*γ* function that contributes to a pathological program of gene expression. Lipidomic analysis suggests that this defect results in part from reduced amounts of the endogenous PPAR*γ*-ligand, 15-keto-prostaglandin E_2_.

## 6. Conclusions

**Figure 3:**
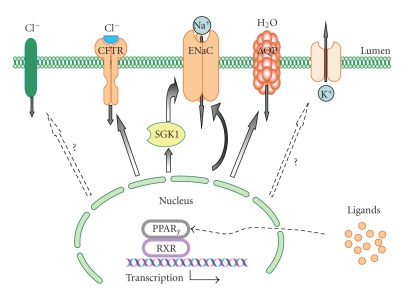
Schematic illustration of the proposed role for PPAR*γ* in the renal collecting duct. PPAR*γ* activation by TZDs or endogenous ligands regulate various epithelial channels/transporters including: (1) epithelial Na^+^ channel (ENaC) either directly or via the Serum- and Glucocorticoid-Regulated Kinase 1 (SGK1); (2) cystic fibrosis transmembrane regulator (CFTR); and (3) AQP water channels. Involvement of PPAR*γ* in the control of ROMK K^+^ and CLC Cl^−^ channels is still unclear. Furthermore, role of PPAR*γ* in the regulation of paracellular transport, and Na-K-ATPase is also proposed (not shown on this scheme).

Here we summarize the current knowledge of PPAR*γ*-dependent regulation of ENaC activity and ENaC-mediated sodium reabsorption. This regulation is clearly not completely understood and requires further studies. Differences in these reported findings may be for example due to the dose and time-course of treatment, species or expression system, and/or the possibility that some studies are examining total protein pools and others mRNA levels. Even *in vivo* studies have their limitations. Additional studies are required to sort out the exact contribution of PPAR*γ* to ENaC regulation. Figure 3 details a possible model for PPAR*γ* regulation of epithelial transport through ENaC-dependent and independent mechanisms in the collecting duct. Thus, the exact mechanism by which PPAR*γ* agonists induce fluid retention is not completely clear but is likely to be multifactorial. It is undoubted that ENaC is not the sole channel responsible for fluid retention and sodium reabsorption in response to PPAR*γ* agonists. Other channels and transporters such as NSC in IMCD, CFTR, Na-K-ATPase, NHE3, and NKCC are most likely also involved in this process. However, a clear account of these proteins in water-electrolyte homeostasis during TZD therapy is still needed. Furthermore, a role of PPAR*α* agonists and RXR is also unclear and requires additional studies.
